# Anxiolytic Effects of Herbal Ethanol Extract from *Gynostemma pentaphyllum* in Mice after Exposure to Chronic Stress

**DOI:** 10.3390/molecules18044342

**Published:** 2013-04-12

**Authors:** Hyun Sook Choi, Ting Ting Zhao, Keon Sung Shin, Seung Hwan Kim, Bang Yeon Hwang, Chong Kil Lee, Myung Koo Lee

**Affiliations:** 1 Department of Pharmacy, College of Pharmacy and Research Center for Bioresource and Health, Chungbuk National University, Cheongju 361-763, Korea; E-Mails: 6151494@hanmail.net (H.S.C); t-t-zho@hotmail.com (T.T.Z); wannaks@hanmail.net (K.S.S); byhwang@chungbuk.ac.kr (B.Y.H); cklee@chungbuk.ac.kr (C.K.L); 2 Institute of Sports Science, College of Physical Education, Kyunghee University, Youngin 449-701, Korea; E-Mail: barkleyk34@khu.ac.kr

**Keywords:** *Gynostemma pentaphyllum*, chronic stress, anxiety disorders, elevated plus-maze, marble burying, dopamine, serotonin, corticosterone, c-Fos

## Abstract

In this study, the effects of herbal ethanol extracts of *Gynostemma pentaphyllum* (GP-EX), on chronic electric footshock (EF) stress-induced anxiety disorders were investigated in mice, which were orally treated with GP-EX (30 mg/kg and 50 mg/kg) once a day for 14 days, followed by exposure to EF stress (2 mA, with an interval and duration of 10 s for 3 min). After the final exposure to EF stress, the elevated plus-maze and marble burying tests were performed, and the levels of dopamine and serotonin in the brain, the serum levels of corticosterone, and the expression of c-Fos in the paraventricular nuclei (PVN) were determined. Treatment with GP-EX (30 mg/kg and 50 mg/kg) significantly recovered the number of entries into open arms and time spent on open arms, which was reduced by chronic EF stress. GP-EX (30 mg/kg and 50 mg/kg) also reduced the number of marbles buried, which was increased by chronic EF stress. In addition, electric EF stress significantly decreased the levels of dopamine and serotonin in the brain, which was recovered by treatment with GP-EX (30 mg/kg and 50 mg/kg). The serum levels of corticosterone, which were markedly increased by chronic EF stress, were reduced by treatment with GP-EX (30 mg/kg and 50 mg/kg). Chronic EF stress-induced increases in c-Fos expression were also markedly reduced by GP-EX (30 mg/kg and 50 mg/kg) in the PVN. These results suggest that GP-EX shows anxiolytic functions, determined by the elevated plus-maze and marble burying tests, which are mediated by modulating the activity of dopamine and serotonin neurons as well as the expression of c-Fos in the brain, and the serum levels of corticosterone. Clinical trials of herbal GP-EX and its bioactive components need further investigation.

## 1. Introduction

Acute and chronic stresses are characterized by the physiological changes that occur in response to novel or threatening stimuli. The neuroendocrine damages in response to acute and chronic stresses are mediated by both the sympathetic nerve system and the hypothalamus-pituitary-adrenal (HPA) axis, which lead to the regulation of the release of dopamine, norepinephrine, serotonin, glutamate, and corticotropin-releasing and adrenocorticotropic hormones in central nervous system (CNS) and the secretion of glucocorticoids in plasma [1]. Chronic stress has been linked to the pathophysiology of mood including anxiety disorders and depression [2]. Beside neuroendocrine release, including that of dopamine, serotonin and glucocorticoids, c-Fos expression, one of the immediate early genes, is increased by acute and chronic stresses within the paraventricular nuclei (PVN) in the hypothalamus of mice and rats, as a marker of altered neuronal responses [3,4].

Chronic stress and chronic stress-induced anxiety disorders have become increasingly more important public health concerns in recent years. However, the ranges of available pharmacotherapy for the treatment of anxiety disorders induced by chronic stress are limited and suboptimal with regard to efficacy and tolerability [5]. 

A number of chronic stress models, including electric footshock (EF) stress, forced swimming, noise stimulus and immobilization, have been employed to induce anxiety disorders in the past decades [6]. In addition, elevated plus-maze is a widely used test, which is based on the natural aversion of rodents to heights and open spaces, and which has been validated to be suitable for the assessment of anxiety disorders for both mice and rats [7]. As another characteristic behavior of the anxiety state of animals, the defensive nature of marble burying has been used as a screening model for the detection of anxiolytics [7,8].

*Gynostemma pentaphyllum* (GP, Cucurbitaceae) has been used as an herbal tea containing various bioactive gypenoside derivatives and flavonoids, and has been found to have pharmacological functions against diabetes, fatigue, hyperlipidemia, immunity, oxidative stress and tumors [9]. Recently, the ethanol extract from GP (GP-EX) has been reported to have anti-stress properties, by improving the loss of body weight and the reduction of grip strength which was induced by chronic EF stress [10] as well as an immunomodulatory function in mice [11,12]. 

In addition, GP-EX has protective effects against neurotoxicity by reducing tyrosine hydroxylase neuron cell death, and by normalizing dopamine levels in the 6-hydroxydopamine (6-OHDA) lesion rat model of Parkinson’s disease (PD) [13]. The gypenoside-rich fraction also shows neuroprotective effects in the 1-methyl-4-phenyl-1,2,3,6-tetrahydropyridine-induced mouse model of PD [14].

In this study, in order to further define the anxiolytic functions of GP-EX, the neuropharmacological effects of GP-EX on chronic EF stress-induced anxiety disorders were investigated by examining the behavioral evaluation, using the elevated plus-maze and marble burying tests, and determining the influences on the levels of dopamine and serotonin in the brain, the serum levels of corticosterone, and the expression of c-Fos in the PVN, using a mouse animal model system.

## 2. Results

### 2.1. Effects of GP-EX on Elevated Plus-Maze

**Figure 1:**
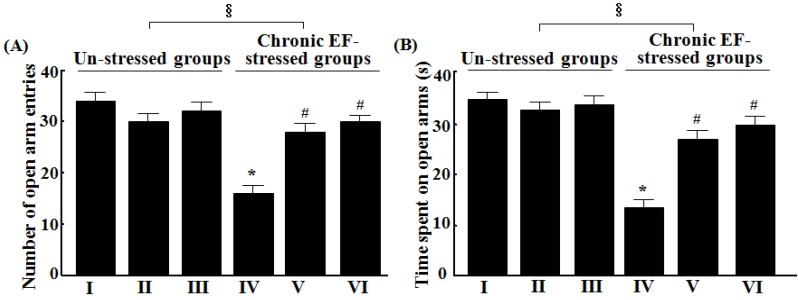
Effects of GP-EX on the number of open arm entries (**A**) and time spent on open arms (**B**) in the elevated plus-maze test in mice. (I) the control groups (0.9% saline-treated groups), (II) GP-EX (30 mg/kg)-treated groups, (III) GP-EX (50 mg/kg)-treated groups, (IV) chronic EF-stressed groups, (V) GP-EX (30 mg/kg)-treated groups in Group IV, (VI) GP-EX (50 mg/kg)-treated groups in Group IV. Mice were orally treated with GP-EX (30 mg/kg and 50 mg/kg) or saline (0.9%) once a day for 14 days. Mice were also exposed to EF stimuli (2 mA, with an interval and duration of 10 s for 3 min) for chronic stress for 14 days. The elevated plus-maze test was performed as described under the Experimental section. The results are expressed as means ± S.E.M. for 8–10 animals per group. * *p* < 0.05 compared with the control groups; # *p* < 0.05 compared with the chronic EF-stressed groups (one-way ANOVA followed by Tukey’s test); § *p* < 0.05 (A, f = 9.490; B, f = 17.668) compared with the un-stressed groups (two-way ANOVA followed by Tukey’s test).

Treatment with GP-EX (30 mg/kg and 50 mg/kg) once a day for 14 days did not alter the results of the elevated plus-maze test in terms of open arm entries and time spent on open arms compared with the control groups (Figure 1). In contrast, the number of open arm entries and time spent on open arms were decreased to ca. 47.1% and 38.8% by chronic EF stress, as compared with the control groups (n = 8, *p* < 0.05). However, treatment with GP-EX at 30 mg/kg and 50 mg/kg for 14 days recovered to ca. 82.2% and 88.0% the number of open arm entries, respectively, which had been reduced by chronic EF stress (n = 8, *p* < 0.05). The reduction in time spent on open arms due to chronic EF stress was also recovered to ca. 77.6% and 85.7% that of the control groups by GP-EX at 30 and 50 mg/kg, respectively, as compared with the chronic EF-stressed groups (n = 8, *p* < 0.05) (Figure 1). 

### 2.2. Effects of GP-EX on Marble Burying

**Figure 2:**
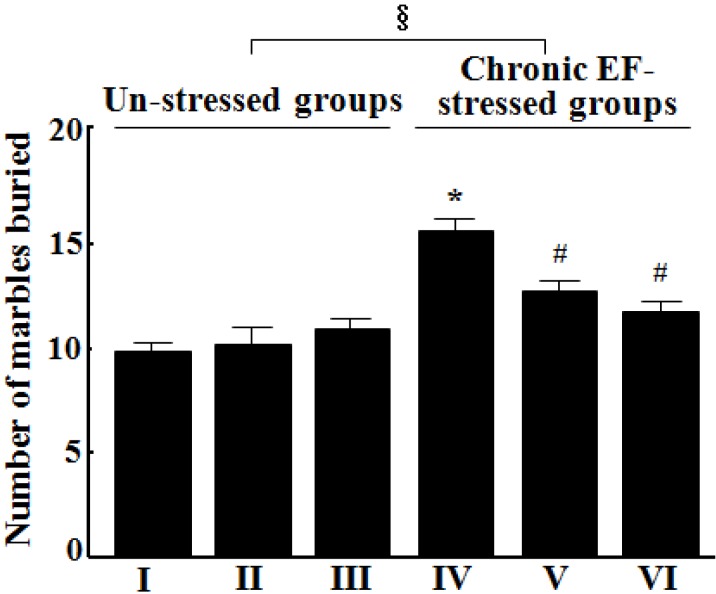
Effects of GP-EX on the number of marbles buried in the marble burying test in mice. (I) the control groups (0.9% saline-treated groups), (II) GP-EX (30 mg/kg)-treated groups, (III) GP-EX (50 mg/kg)-treated groups, (IV) chronic EF-stressed groups, (V) GP-EX (30 mg/kg)-treated groups in Group IV, (VI) GP-EX (50 mg/kg)-treated groups in Group IV. Mice were orally treated with GP-EX (30 mg/kg and 50 mg/kg) or saline (0.9%) once a day for 14 days. Mice were also exposed to EF stimuli (2 mA, with an interval and duration of 10 s for 3 min) for chronic stress for 14 days. The marble burying test was performed as described under the Experimental section. The results are expressed as means ± S.E.M. for 8–10 animals per group. * *p* < 0.05 compared with the control groups; # *p* < 0.05 compared with the chronic EF-stressed groups (one-way ANOVA followed by Tukey’s test); § *p* < 0.05 (f = 13.697) compared with the un-stressed groups (two-way ANOVA followed by Tukey’s test).

Treatment with GP-EX (30 mg/kg and 50 mg/kg, 14 days) had no effect on the number of marbles buried (Figure 2). However, the number of marbles buried was increased to ca. 160.2% (by 60.2%) among chronic EF stress-induced groups, compared with that of control groups (n = 10, *p* < 0.05) (Figure 2). Treatment with GP-EX at 30 mg/kg and 50 mg/kg significantly reduced this increase to ca. 28.1% and 24.3% that of the control groups, respectively (n = 10, *p* < 0.05), which indicated a dose-dependent response (Figure 2).

### 2.3. Effects of GP-EX on the Levels of Dopamine in the Brain

**Figure 3:**
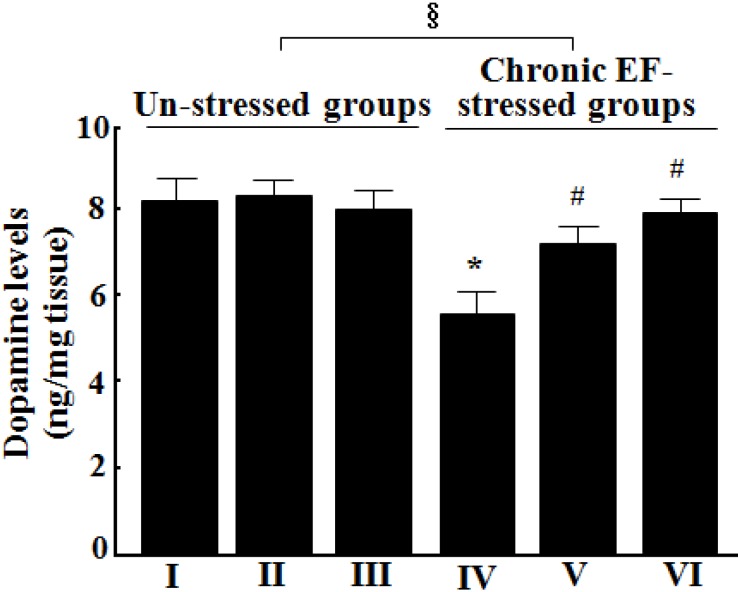
Effects of GP-EX on the levels of dopamine in the brain. (I) the control groups (0.9% saline-treated groups), (II) GP-EX (30 mg/kg)-treated groups, (III) GP-EX (50 mg/kg)-treated groups, (IV) chronic EF-stressed groups, (V) GP-EX (30 mg/kg)-treated groups in Group IV, (VI) GP-EX (50 mg/kg)-treated groups in Group IV. Mice were orally treated with GP-EX (30 mg/kg and 50 mg/kg) or saline (0.9%) once a day for 14 days. Mice were also exposed to EF stimuli (2 mA, with an interval and duration of 10 s for 3 min) for chronic stress for 14 days. The brains were removed, and the levels of dopamine were determined by an HPLC method. The results are expressed as means ± S.E.M. for 8–10 animals per group. * *p* < 0.05 compared with the control groups; # *p* < 0.05 compared with the chronic EF-stressed groups (one-way ANOVA followed by Tukey’s test); § *p* < 0.05 (f = 6.196) compared with the un-stressed groups (two-way ANOVA followed by Tukey’s test).

As shown in Figure 3, GP-EX (30 mg/kg and 50 mg/kg) treatment did not affect the levels of dopamine in the brain, as compared with that of the control groups. The observed levels were comparable to those observed in a previous study [15]. In this study, the levels of dopamine were reduced to ca. 68.4% by chronic EF stress (n = 8, *p* < 0.05). However, the levels of dopamine reduced by chronic EF stress were recovered to ca. 87.8% and 96.5% that of the control groups, by treatment with GP-EX at 30 mg/kg and 50 mg/kg for 14 days, respectively (n = 8, *p* < 0.05) (Figure 3). 

### 2.4. Effects of GP-EX on the Levels of Serotonin in the Brain

**Figure 4:**
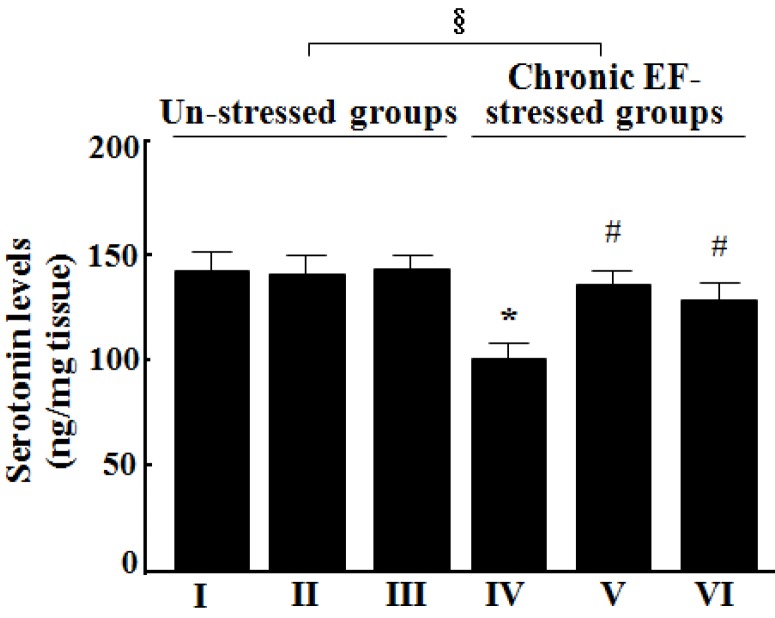
Effects of GP-EX on the levels of serotonin in the brain. (I) the control groups (0.9% saline-treated groups), (II) GP-EX (30 mg/kg)-treated groups, (III) GP-EX (50 mg/kg)-treated groups, (IV) chronic EF-stressed groups, (V) GP-EX (30 mg/kg)-treated groups in Group IV, (VI) GP-EX (50 mg/kg)-treated groups in Group IV. Mice were orally treated with GP-EX (30 mg/kg and 50 mg/kg) or saline (0.9%) once a day for 14 days. Mice were also exposed to EF stimuli (2 mA, with an interval and duration of 10 s for 3 min) for chronic stress for 14 days. The brains were removed, and the levels of serotonin were determined by HPLC analysis. The results are expressed as means ± S.E.M. for 8–10 animals per group. * *p* < 0.05 compared with the control groups; # *p* < 0.05 compared with the chronic EF-stressed groups (one-way ANOVA followed by Tukey’s test); § *p* < 0.05 (f = 29.392) compared with the un-stressed groups (two-way ANOVA followed by Tukey’s test).

The levels of serotonin in the brain were decreased to ca. 70.3% that of the control groups by exposure to chronic EF stress (n = 8, *p* < 0.05) (Figure 4). However, the level of serotonin was recovered to ca. 95.1% and 90.9% that of the control groups by treatment with GP-EX at 30 mg/kg and 50 mg/kg, respectively (n = 8, *p* < 0.05) (Figure 4).

### 2.5. Effects of GP-EX on the Levels of Corticosterone in the Serum

**Figure 5:**
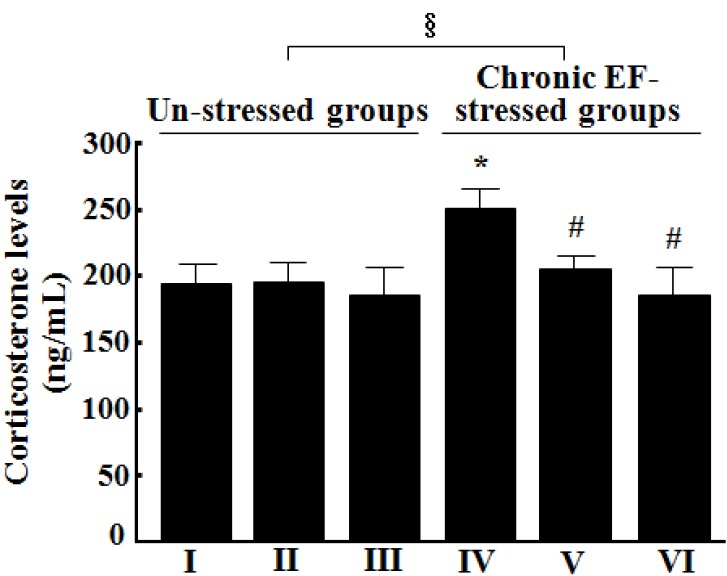
Effects GP-EX on the serum levels of corticosterone. (I) the control groups (0.9% saline-treated groups), (II) GP-EX (30 mg/kg)-treated groups, (III) GP-EX (50 mg/kg)-treated groups, (IV) chronic EF-stressed groups, (V) GP-EX (30 mg/kg)-treated groups in Group IV, (VI) GP-EX (50 mg/kg)-treated groups in Group IV. Mice were orally treated with GP-EX (30 mg/kg and 50 mg/kg) or saline (0.9%) once a day for 14 days. Mice were also exposed to EF stimuli (2 mA, with an interval and duration of 10 s for 3 min) for chronic stress for 14 days. The blood samples were collected on the last day of the behavioral tests, and the serum levels of corticosterone were determined by an enzyme-linked immunosorbent assay kit. The results are expressed as means ± S.E.M. for 8–10 animals per group. * *p* < 0.05 compared with the control groups; # *p* < 0.05 compared with the chronic EF-stressed groups (one-way ANOVA followed by Tukey’s test); § *p* < 0.05 (f = 10.732) compared with the un-stressed groups (two-way ANOVA followed by Tukey’s test).

Treatment with GP-EX at 30 mg/kg and 50 mg/kg once a day for 14 days did not alter the serum levels of corticosterone, as compared with the control groups (n = 8) (Figure 5). In contrast, the levels of corticosterone in the serum were increased to ca. 129.3% that of the control groups by exposure to chronic EF stress (n = 8, *p* < 0.05). However, the released levels of corticosterone by chronic EF stress were reduced to ca. 105.2% and 95.4% that of the control groups by treatment with GP-EX at 30 mg/kg and 50 mg/kg for 14 days (n = 8, *p* < 0.05) (Figure 5).

### 2.6. Effects of GP-EX on c-Fos-immunoreactive Cells in the PVN

**Figure 6:**
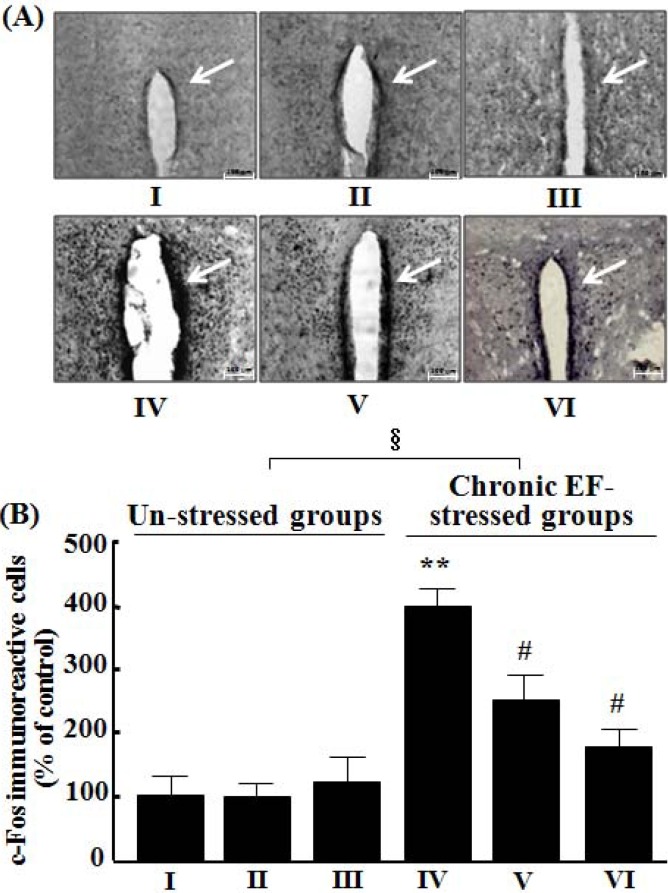
Representative photographs illustrating the effects of GP-EX on c-Fos-immunoreactive cells in the paraventricular nuclei (PVN) (**A**) and the number of c-Fos-immunoreactive cells in the PVN (**B**). (I) the control groups (0.9% saline-treated groups), (II) GP-EX (30 mg/kg)-treated groups, (III) GP-EX (50 mg/kg)-treated groups, (IV) chronic EF-stressed groups, (V) GP-EX (30 mg/kg)-treated groups in Group IV, (VI) GP-EX (50 mg/kg)-treated groups in Group IV. Mice were orally treated with GP-EX (30 mg/kg and 50 mg/kg) or saline (0.9%) once a day for 14 days. Mice were also exposed to EF stimuli (2 mA, with an interval and duration of 10 s for 3 min) for chronic stress for 14 days. (**A**) Immunoblots of lysates from the brain were probed with c-Fos antibodies, and the total c-Fos-immunoreactive cells were measured as described under the Experimental section; (**B**) The number of c-Fos-immunoreactive cells was counted in the PVN and was expressed as a percentage of the control groups. The number of c-Fos-immunoreactive cells of the control groups was 25 ± 6 cells per section. The arrow indicates nucleus of c-Fos positive neurons. Scale bar is 100 μm. The results are expressed as means ± S.E.M. (n = 7). * *p* < 0.05 compared with the control groups; # *p* < 0.05 compared with the chronic EF-stressed groups (one-way ANOVA followed by Tukey’s test); § *p* < 0.05 (f = 127.355) compared with the un-stressed groups (two-way ANOVA followed by Tukey’s test).

The expression of c-Fos in the PVN was markedly increased to ca. 4.1-fold that of the control groups by exposure to chronic EF stress for 14 days (Figure 6A,B). However, the expression of c-Fos was reduced by treatment with GP-EX (30 mg/kg and 50 mg/kg) in the PVN (Figure 5A). The number of c-Fos-immunoreactive nuclei in PVN, which was increased by chronic EF stress, was significantly reduced to ca. 2.4-fold and 1.3-fold that of the control groups by treatment with GP-EX at 30 mg/kg and 50 mg/kg, respectively (n = 7, *p* < 0.05) (Figure 6).

## 3. Discussion

GP-EX has an ameliorating function on the loss of body weight and the reduction of grip strength, induced by chronic EF stress [10]. GP-EX also has a significant protective function on chronic EF stress-induced anxiety, as monitored by observation of ambulatory locomotor activity [15], which is the classical behavioral method for screening anxiety disorders. In this study, for the further precise anxiolytic functions of GP-EX, the effects of GP-EX on chronic EF stress-induced anxiety disorders were investigated by evaluating the elevated plus-maze and marble burying tests, as well as the levels of dopamine, serotonin and corticosterone, and the expression of c-Fos, in mice as an animal model system.

The elevated plus-maze test is used to evaluate anxiety disorders, utilizing the natural fear of rodents for open and elevated places [7,16]. Mice normally prefer to spend much of their allotted time in the closed arms of the model system (as a more secure location), reflecting an aversion toward the open arms, that is generated by a fears of the open spaces [7,16]. The marble burying test was also used to measure anxiety-like behavior and obsessive-compulsive behavior [8]. The suppression of spontaneous burying of harmless objects by rodents has been used as an index of anxiolytic drug activity [17]. In addition, the anxiety behavior in the elevated plus-maze test is expressed as a passive avoidance in response to a potential threat. In contrast, the defensive burying behavior in the marble burying test represents an active coping strategy in response to a discrete threat including the predictive validity for anxiety [18].

In our study, treatment with GP-EX at 30 mg/kg and 50 mg/kg significantly recovered the number of entries into open arms and time spent on open arms, which was reduced by chronic EF stress (Figure 1). Closed arm entries, a measure of motor activity in the elevated plus-maze test, were slightly decreased by GP-EX, but this reduction was not significant. In addition, the total distance traveled and the number of total arm entries in the elevated plus-maze test were slightly reduced by chronic EF stress as compared with the control groups, but it was not significant. However, the ratios of the number of open arm entries to the total arm entries were significantly reduced in the chronic EF-stressed groups as compared with the control groups (data not shown). This ratio is considered to be related to the level of anxiety in the test animals**.** GP-EX (30 mg/kg and 50 mg/kg) also reduced the number of marbles buried, which was increased by chronic EF stress (Figure 2). Previously, the spontaneous locomotor activity in mice, which was decreased by chronic EF stress, was shown to be increased by treatment with GP-EX (30 mg/kg) [15]. These results indicate that GP-EX exhibits anxiolytic-like functions without depressing spontaneous locomotor activity, which has been examined in this study through evaluation of both the elevated plus-maze and marble burying tests. It is also suggested that GP-EX relieves the anxiety symptoms induced by both the potential and discrete threats in mice.

Exposure to chronic stress induces anxiety disorders at the relatively early-time periods and then induces depression [15], which is related at least partially to the decreased levels of dopamine and serotonin in the brain [19]. It is also reported that dopamine depletion in the brain increases anxiety disorders in response to a discrete threat stimuli in mice [18]. Acute and chronic stresses activate the HPA system, which can result in an increase in levels of corticosterone in the blood compared to the unstressed control groups: the levels of corticosterone induced by acute stress are higher than those induced by chronic stress [20]. An increase in the levels of glucocorticoid hormones is also known to decrease serotonin metabolism in the CNS [21]. In addition, serotonin receptor activation increases anxiolytic-like behavioral responses, as measured by the elevated plus-maze test [22]. The number of marbles buried is reduced by benzodiazepine receptors agonist (diazepam) and serotonin reuptake inhibitors (fluoxetine and clomipramine) [23]. Spontaneous locomotor activity is also reduced by dopamine receptor blocking agents [24].

In our study, electric EF stress significantly decreased the levels of dopamine and serotonin in the whole brain, which was recovered by treatment with GP-EX (30 mg/kg and 50 mg/kg) for 14 days (Figure 4). The serum levels of corticosterone, which were markedly increased by chronic EF stress, were also reduced by treatment with GP-EX (30 mg/kg and 50 mg/kg) for 14 days (Figure 5). These results, as well as those of the behavioral tests, suggest anxiolytic functions of GP-EX, as mediated by modulation of dopamine and serotonin neuron activity in the brain. 

Chronic stress induces changes in PVN peptide expression related to glucocorticoids and GABA receptor expression [25,26]. Fos family members have been mainly identified as Fos, FosB, Fra-1 and Fra-2, and ΔFosB, the truncated form of FosB, which is induced in response to repeated social defeat stress, drug abuse and natural reward [27,28]. It is also reported that FosB-like immunoreactivity as seen for c-Fos in the brain is increased by acute stress, and the FosB levels remain high after chronic stress [29,30]. In addition, the c-Fos expression in PVN, which is associated with HPA axis, is increased by acute and chronic stress in mice and rats [3,4].

Exposure to chronic EF stress increased c-Fos expression in the PVN regions of mice (Figure 6). In contrast, chronic EF stress-induced increases in c-Fos expression were markedly reduced by GP-EX (30 mg/kg and 50 mg/kg) in the dopaminergic terminals of PVN (Figure 6A,B), suggesting that the modulation of c-Fos expression by GP-EX plays a role in the protective functions against chronic stress.

Stressful stimuli induce the production of reactive oxygen species (ROS) and increase the release of catecholamines and glucocorticoids [31], which reduce the function of immune systems [11]. The exposure to chronically repetitive stress also reduces dopamine levels in the rat brain, leading to a decrease in spontaneous locomotor activity [32].

GP-EX shows an immunomodulatory activity by preventing dexamethasone-induced immunosuppression [11]. GP-EX has protective effects against 6-OHDA-induced neurotoxicity by reducing tyrosine hydroxylase neuronal cell death in the 6-OHDA lesioned rat model of PD [13] and butanol extracts of GP also protect against the chronic EF stress, which reduces L-DOPA-induced neuronal cell death in 6-OHDA-lesioned rats treated with L-DOPA [33]. These results suggest that the protective functions of GP-EX on chronic EF stress-induced neurotoxicity are mediated by modulating the ROS formation and immune systems in rodents.

GP-EX at doses of 10–50 mg/kg/day for 28 days does not show adverse effects in rats [10,11]. The water extract (750 mg/kg) of GP does not produce any significant toxicity in rats during a 6-month period of treatment [34]. Butanol fractions from GP-EX at 30 mg for 28 days do not also exhibit adverse effects, such as weight loss, diarrhea, vomiting and death [33]. Bioactive components have been isolated from GP-EX, and have been identified as gypenoside derivatives, including gynosaponin TN-1, gynosaponin TN-2, gypenoside XLV and gypenoside LXXIV [9], which has been found to have protective effects on oxidative stress induced by glutamate-induced neurotoxicity [35]. 

Several natural products have been reported to have an anti-stress activity. Lobeline, obtained from *Lobelia inflate* Linn, has anxiolytic potential and nAChR antagonistic effects in C57BL/6J mice [36]. *Schisandra* lignan extracts exhibits an anti-stress activity by modulating the hyperactive HPA axis [37]. *Bacopa monnieri* also exhibits the normalization of stress-induced alteration in plasma corticosterone and the levels of serotonin, dopamine and norepinephrine in the cortex of the brain [19]. The comparative neuropharmacological functions on anxiety disorders may need to be studied prior to clinical applications. 

## 4. Experimental

### 4.1. Chemicals

Dopamine, norepinephrine, serotonin, 5-hydroxyindole acetic acid (HIAA) were obtained from Sigma (St. Louis, MO. USA). The corticosterone kit was purchased from USCN Life Sci. (E0504m, Wuhan, China). c-Fos antibody was obtained from Santa Cruz Biotechnology (Santa Cruz, CA, USA). Anti-mouse IgG, vectastain diaminobenzidine (DAB) and avidin/biotin complex (ABC) kits were purchased from Vector Laboratories, Inc. (Burlingame, CA, USA). All other chemicals were of analytical grade.

### 4.2. Preparation of GP-EX

GP was obtained from Wonkwang Food Manufacturing Co. (Geochang, Korea), and a voucher specimen of the herbal leaves of GP was deposited at the herbarium of the College of Pharmacy, Chungbuk National University (Cheongju, Korea). The air-dried leaves of GP (10 kg) were extracted with ethanol (20 L, 80%, v/v) at 70–80 °C for 24 h, and the ethanol extracts were then evaporated to dryness (GP-EX, 1.05 kg; yield, 10.5%, w/w). The main components of GP-EX including gypenosides and ombuoside were identified to standardize the ethanol extracts [9,13].

### 4.3. Animals

Mice (ICR, male, 20–25 g) were purchased from Samtako Co. (Animal Breeding Center, Osan, Korea). Animals were housed two per cage in a temperature controlled environment with a 12-h light/dark cycle (lights on at 07:00) and with *ad libitum* access to standard mouse food and water. This study was performed in accordance with the guidelines for the care and use of laboratory animals of Chungbuk National University Laboratory Animal Research Center (approval number: CBNU-481-12-01).

### 4.4. Experimental Design

Mice were divided randomly into six groups, with each group containing 8–10 animals. GP-EX (30 mg/kg and 50 mg/kg), which was freshly prepared every day with water, was administered orally once a day ca. 2 h before the exposure of EF stress for 14 days. 

Group I was the control groups, and received saline (0.9%). Group II and Group III were the GP-EX-treated groups, and were treated with GP-EX at 30 mg/kg (Group II) and 50 mg/kg (Group III) for 14 days.Group IV was the chronic EF-stressed groups. Group V and Group VI were the GP-EX-treated groups, and received EF stimuli for 14 days, as well as being treated with GP-EX at 30 mg/kg (Group V) and 50 mg/kg (Group VI). The experiments were performed in the independent group of animals. On the final day (day 14), after the final administration of each treatment, all experimental groups were subjected to the elevated plus-maze or marble burying test, and the mice were then anaesthetized and sacrificed to obtain brain tissues and serum for biochemical and immuno-histochemical analyses.

### 4.5. The Exposure to Chronic EF Stress

The mice were placed individually in the electrified shock chamber for the exposure to chronic stress and they received unavoidable EF stimuli (intensity, 2 mA; interval and duration, 10 s; periods, 3 min) at 14:00 every day for 14 days using a shock generator (Seil Electric Co., Taejeon, Korea) [10].

### 4.6. The Elevated Plus-Maze Test

The elevated plus-maze apparatus consists of four arms (30 cm × 5 cm) and two closed arms of the same size, with 16-cm-high black walls elevated 45 cm above the floor. The two arms of each type were arranged on opposite sides to each other. The open and closed arms were connected via a central square (5 cm × 5 cm) to form a plus sign. During test sessions, two incandescent lights (40 W each) provided sufficient illumination of the maze area. Testing commenced upon placing a mouse on the central platform of the maze, facing an open arm. An arm entry was defined as all four paws having crossed the dividing line between an arm and the central area. During a 5 min test period, the number of entries and time spent on the open arms were recorded by a video camera connected to a video-tracking system SMART (Panlab S.I., Barcelona, Spain) [38].

### 4.7. The Marble Burying Test

The marble burying test was placed in a cage measuring 33 × 21 × 19 cm (l × w × h), containing bedding that was 5 cm in depth, with twenty-five marbles 2 cm in diameter, arranged in two rows along the same short wall of the cage [8]. Testing was conducted for a 30-min period under red light and white noise. After a 30-min exposure to the marbles, mice were removed and the number of marbles buried was recorded. Marbles were considered to be buried if at least half of their surface was covered with bedding [8,18].

### 4.8. Measurement of Dopamine Levels

The whole brains were removed immediately and brain tissues were homogenized in perchloric acid (1 M, 300 μL), and the homogenates were centrifuged at 12,000 × *g* at 4 °C for 20 min. The supernatants were filtered using pore filters (Millex-GV, 0.45 μm, Waters, Milford, MA, USA) and the filtrate (100 μL) was injected into an HPLC system with electrochemical detection [39]. The results were expressed in terms of ng/mg tissue.

### 4.9. Measurement of Serotonin Levels

The whole brain tissues were homogenized in trichloroacetic acid (0.3 M, 500 μL) and HIAA (300 pmol, internal standard). The homogenates were immediately centrifuged at 12,000 × *g* at 4 °C for 20 min. The supernatants were then filtered using pore filters (Millex-GV, 0.45 μm, Waters) and the filtrate (100 μL) was injected into an HPLC system with fluorescence detection [40]. The results were expressed in terms of ng/mg tissue.

### 4.10. Measurements of Corticosterone Levels

Blood was collected from the heart of sacrificed mice, and was then centrifuged at 12,000 × *g* at 4 °C for 15 min to obtain a serum. The serum levels of corticosterone were assessed using an enzyme-linked immunosorbent assay kit (USCN Life Sci., E0540m, Wuhan, China). 

### 4.11. Immunohistochemistry of c-Fos

After the behavioral test, mice were anaesthetized (Zoletil, 50 mg/kg, i.p., Virbac, Carros, France) and transcardially perfused with saline, followed by a paraformaldehyde solution (4% in 0.1 M phosphate buffered saline, pH 7.4), after which the brains were removed. Coronal brain sections (35 μm) at the level of the PVN were processed for c-Fos immunocytochemistry using a polyclonal rabbit anti-c-Fos primary antibody (1:20,000), a biotinylated goat anti-rabbit secondary antibody (1:200; Vector Laboratories) and an ABC kit procedure with DAB kit (Vector Laboratories) as chromogens according to protocols. The PVN cells containing a nuclear brown-black reaction product were considered to be c-Fos-positive cells, and they were counted using a computerized image analysis system (Axiovision software, Carl Zeiss MicroImaging, GmbH, Jena, Germany) using a microscope (Zeiss Axiophot, Carl Zeiss MicroImaging) (100× magnification). 

### 4.12. Statistical Analysis

Data were analyzed with a one-way analysis of variance (ANOVA) followed by Tukey’s test for evaluating the dose-dependent effects of GP-EX. Two-way ANOVA followed by Tukey’s test was also used to evaluate the effects of GP-EX on chronic EF stress. All data were expressed as means ± S.E.M. with *p* values of <0.05 being considered to be statistically significant.

## 5. Conclusions

In this study, in the control groups, treatment with GP-EX (30 mg/kg and 50 mg/kg) did not alter the numbers of open arm entries and marbles buried in the behavioral tests, and also did not show any interaction between the control and GP-EX-treated groups. In contrast, GP-EX (30 mg/kg and 50 mg/kg) showed the ameliorating functions on anxiety disorders induced by chronic EF stress in mice. The anxiolytic functions of GP-EX on the results of the elevated plus-maze and marble burying tests occur due to modulation of the dopamine and serotonin neuron activity, as well as the expression of c-Fos in the brain, and the serum levels of corticosterone, which was suggested to be associated with the PVN regions and HPA axis. These data contribute to the application and development of GP-EX though herbal GP-EX and its bioactive components need to be studied further. The precise neurochemincal mechanisms of GP-EX on anxiety disorders induced by the potential and discrete threat stimuli also need to be investigated prior to clinical trials.
